# A Bead-Based Multiplexed Immunoassay to Evaluate Breast Cancer Biomarkers for Early Detection in Pre-Diagnostic Serum

**DOI:** 10.3390/ijms131013587

**Published:** 2012-10-22

**Authors:** Annemieke W. J. Opstal-van Winden, Wendy Rodenburg, Jeroen L. A. Pennings, Conny T. M. van Oostrom, Jos H. Beijnen, Petra H.M. Peeters, Carla H. van Gils, Annemieke de Vries

**Affiliations:** 1 Julius Center for Health Sciences and Primary Care, University Medical Center Utrecht, Universiteitsweg 100, 3584 CG Utrecht, The Netherlands; E-Mails: a.v.winden@nki.nl (A.W.J.O.-W.); p.h.m.peeters@umcutrecht.nl (P.H.M.P.); 2 Department of Pharmacy and Pharmacology, The Netherlands Cancer Institute and Slotervaart Hospital, Louwesweg 6, 1066 EC Amsterdam, The Netherlands; E-Mail: jos.beijnen@slz.nl; 3 Laboratory for Health Protection Research, National Institute for Public Health and the Environment (RIVM), A. van Leeuwenhoeklaan 9, 3721 MA Bilthoven, The Netherlands; E-Mails: wendy.rodenburg@rivm.nl (W.R.); jeroen.pennings@rivm.nl (J.L.A.P.); conny.van.oostrom@rivm.nl (C.T.M.O.); annemieke.de.vries@rivm.nl (A.V.); 4 Department of Pharmaceutical Sciences, Division of Biomedical Analysis, Section of Drug Toxicology, Faculty of Science, Utrecht University, Sorbonnelaan 16, 3584 CA Utrecht, The Netherlands; 5 Department of Epidemiology, Public Health and Primary Care, Faculty of Medicine, Imperial College, Norfolk Place, London W2 1PG, UK

**Keywords:** biomarkers, breast cancer, detection, pre-diagnostic, serum

## Abstract

This study investigates whether a set of ten potential breast cancer serum biomarkers and cancer antigens (osteopontin (OPN), haptoglobin, cancer antigen 15-3 (CA15-3), carcinoembryonic antigen (CEA), cancer antigen 125 (CA-125), prolactin, cancer antigen 19-9 (CA19-9), α-fetoprotein (AFP), leptin and migration inhibitory factor (MIF)) can predict early stage breast cancer in samples collected before clinical diagnosis (phase III samples). We performed a nested case-control study within the Prospect-EPIC (European Prospective Investigation into Cancer and nutrition) cohort. We examined to what extent the biomarker panel could discriminate between 68 women diagnosed with breast cancer up to three years after enrollment and 68 matched healthy controls (all 56–64 years at baseline). Using a quantitative bead-based multiplexed assay, we determined protein concentrations in serum samples collected at enrollment. Principal Component Analysis (PCA) and Random Forest (RF) analysis revealed that on the basis of all ten proteins, early cases could not be separated from controls. When we combined serum protein concentrations and subject characteristics related to breast cancer risk in the RF analysis, this did not result in classification accuracy scores that could correctly classify the samples (sensitivity: 50%, specificity: 50%). Our findings indicate that this panel of selected tumor markers cannot be used for diagnosis of early breast cancer.

## 1. Introduction

The identification of blood biomarkers for early detection of breast cancer is an important target of research. Mammography, the current routine method for early detection, has a limited sensitivity for the detection of tumors in dense breast tissue [1]. A blood test would be easy to perform and would also omit the imaging-related problem of high breast density [1]. As such, it would also offer potential for younger women, who are now excluded from most breast cancer screening programs, mainly because the prevalence of dense breast tissue is very high in this group.

To date, researchers have identified many substances that seem to be differentially expressed in blood when certain types of cancer are present [2]. These substances, or cancer biomarkers, are mainly tested for use in monitoring, *i.e.*, following whether the cancer is indeed in regression due to the anti-cancer treatment, or monitoring whether the cancer is re-occurring, rather than for early cancer detection. Few of these biomarkers have been tested rigorously in pre-diagnostic serum collected from asymptomatic people. Therefore, the utility of available biomarkers for cancer screening is unknown.

The few biomarkers that are tested for early detection of breast cancer, show limited diagnostic sensitivity and specificity in stand-alone assays [2–12]. A panel of biomarkers may have better predictive performance than individual markers. Since breast cancer is a heterogeneous disease [13], it possibly requires a panel of several biomarkers to allow detection of the different subtypes. Combinations of biomarkers have not been extensively tested for their usefulness in early breast cancer detection. Several studies investigated performance of a panel of ovarian cancer biomarkers, compared to CA-125 alone. Most show improved performance of a panel, but a recent study could not confirm this in phase III samples [14–17].

In the current study we evaluated the value of a panel of potential cancer biomarkers for early detection of breast cancer in human females. To this end, we used samples obtained from asymptomatic individuals before clinical diagnosis—so-called “phase III” specimens—instead of “phase II” specimens obtained from symptomatic individuals at diagnosis [18]. We did this to investigate the true ability to detect a tumor in a subclinical stage.

We composed a panel of potential cancer biomarkers for breast cancer, in order to study their combined predictive value for early breast cancer. Three serum proteins that have been approved by the FDA as biomarkers to monitor chemotherapy in patients with advanced breast cancer were included: cancer antigen 15-3 (CA15-3), carcinoembryonic antigen (CEA) and HER2/neu [2,12]. Another tumor marker, cancer antigen 125 (CA-125), is primarily being used together with transvaginal ultrasound for early detection of ovarian cancer in women with hereditary syndromes [12], but has also been suggested as tumor marker for breast cancer [11]. For the serum markers CA15-3, CEA and CA-125, a relation with breast cancer has been found in previous studies, but by themselves were not discriminative enough [2,3,5–12]. We also included several other cancer antigens that are known biomarkers for multiple cancer types, and that were recently shown to be differentially expressed in serum of breast cancer and control subjects [19–22]. These markers are cancer antigen 19-9 (CA19-9), also known for pancreatic, gastric and colon cancer [2,23], α-fetoprotein (AFP), also known for ovary, testis and liver cancer [2], and migration inhibitory factor (MIF), also known for gastric, prostate, and colon cancer [24–26]. MIF has previously been found to be higher in mammary tumor tissues than normal mammary gland tissue [27]. Furthermore, we included prolactin and leptin. Prolactin has previously been found to be a blood-borne risk marker for breast cancer in postmenopausal women, particularly for estrogen receptor positive (ER+) and progesterone receptor positive (PR+) cancers [28]. Leptin has been shown to be associated with breast cancer risk [29], and has been observed to be differentially expressed in serum of breast cancer and control subjects [30–32].

Over the past decade, a growing number of cancers as encountered in human patients have been modeled in mice [33,34]. Animal studies may help in finding appropriate and valid biomarkers. Animal experiments have a shorter time span than human studies and methods of specimen ascertainment are easier to standardize. Using transgenic mouse models that spontaneously develop mammary tumors mimicking human breast cancer, we previously found two markers, osteopontin (OPN) and haptoglobin, which completely distinguished mammary-tumor bearing mice from non-tumor mice [35]. Others also found these markers to be differentially expressed in blood of breast tumor cases and controls in mice and humans [36–40]. Osteopontin expression in mammary tumor tissue [41–43] and plasma [44,45] is also related with progressing disease. We included osteopontin en haptoglobin in our panel under study as well.

The concentrations of our selected proteins, as listed in Table 1, were assessed with bead-based multiplexed immunoassays. This enabled simultaneous analysis of many potential markers and relatively rapid processing of large sets of sera [46,47]. We investigated the discriminative value of this serum tumor marker panel in a nested case-control study within the Prospect-EPIC (European Prospective Investigation into Cancer and nutrition) cohort [48]. We studied women who were diagnosed with breast cancer up to three years after enrollment in the cohort. The concentrations of the tumor markers in breast cancer patients were measured in pre-diagnostic serum samples collected at enrollment, and compared to those of matched controls who remained healthy. This design creates the unique opportunity to evaluate the discriminative power of this panel of tumor markers for detection of early, asymptomatic stages of breast cancer.

## 2. Results and Discussion

### 2.1. Study Population

Characteristics of the study population are presented in Table 2. About half of both the cases and controls used oral contraceptives in the past, but the cases used them for a longer time period than the controls (median duration 10 and 4.5 years, respectively). Cases were somewhat more often nulliparous (15%) than controls (7%). Among women with children, controls had more children than the cases; 3 and 2 (median), respectively. About half of both the cases and controls had smoked in the past, for about 8 and 4 years (median), respectively. The energy-adjusted intake of alcohol was somewhat higher for controls than for cases; 2.5 and 2.0 g/day, respectively (median). Other characteristics were equally distributed in cases and controls. Characteristics of the serum samples are listed in Table 3. There was no difference between the cases and the controls regarding the sample collection and storage.

The cases were diagnosed with breast cancer after a median time of 21.3 months (inter-quartile range (IQR): 0.7–26.6 months) after enrollment. More than 80% of the cases were affected with an invasive tumor. More than half of the invasive tumors had been diagnosed in Stage I and a quarter in Stage IIA. Only one tumor had been diagnosed in Stage IIIA. The invasive tumors were more or less equally distributed over the three size categories (from 0.1 cm to less than 1 cm, 1 cm to less than 2 cm, 2 cm or more). In 30% of the cases with invasive tumors, lymph nodes were involved. None of the cases was affected with metastases. For seven and nine cases, respectively, the pathologically determined tumor size and lymph node involvement were unknown. For all but two of these cases, we were able to report the clinically determined tumor size and lymph node involvement.

### 2.2. Discriminative Value of Candidate Breast Cancer Markers in Pre-Diagnostic Serum

The quality of the measurements was acceptable since coefficients of variance (CVs) were smaller than 15%. Mean concentrations of CA15-3 and MIF were slightly, but not significantly lower in the cases compared to the controls (CA15-3 (U/mL): 8.48 (95% confidence interval (CI): 7.08–10.15) versus 10.34 (95%CI: 8.72–12.26) (*p* = 0.11) and MIF (pg/mL): 166.5 (95%CI: 141.9–195.4) *versus* 199.7 (95%CI: 165.9–240.4) (*p* = 0.13)). Mean concentrations of prolactin were slightly, but not significantly higher in cases compared to controls (4.79 ng/mL (95%CI: 4.09–5.60) versus 4.03 ng/mL (95%CI: 3.48–4.66) (*p* = 0.10). Concentrations of the other individual markers were not found to be different between cases and controls. The geometric mean concentrations and 95% confidence intervals of all proteins in cases and controls, together with the *p*-values are presented in Table 4.

**Figure 1:**
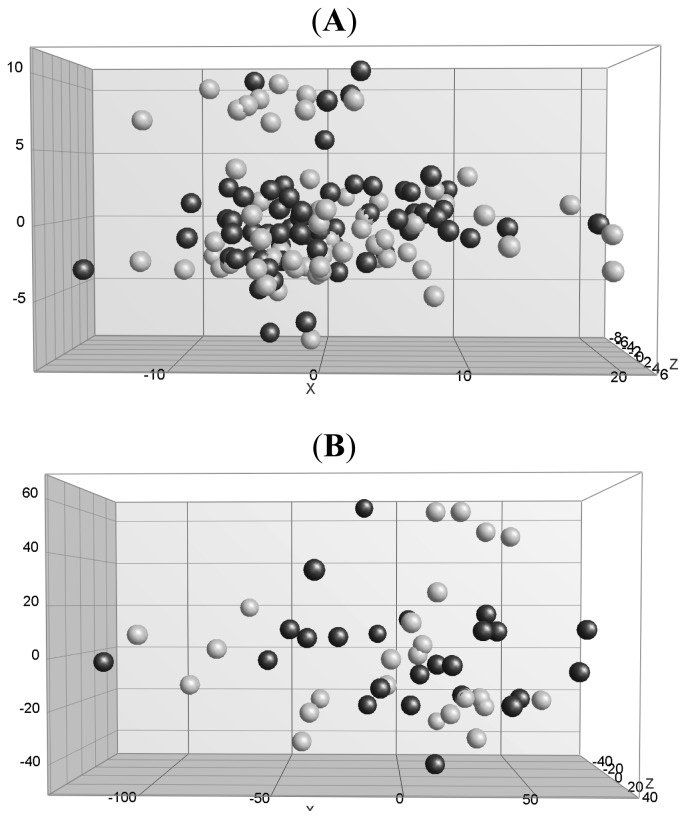
Principal component analysis (PCA) 3D view for protein profiles. Legend: (**A**) for 68 cases (black dots) and 68 controls (gray dots) and (**B**) for the subset consisting of 24 cases and 24 controls. The PCA in both **A** and **B** is based on all ten proteins. The first three components are shown.

To determine whether cases could be distinguished from controls based on a combination of all ten protein markers, principal component analysis (PCA) was performed. Figure 1A represents the PCA 3D view in which the cases and controls are plotted based on the first three principal components explaining most of the variance in the data. The amount of variance explained by the first three components of the total of 9 components was 31.4%, 18.6% and 12.1% respectively. This analysis showed that PCA based on concentrations of all proteins could not distinguish cases from controls. The Random Forests (RF) classification accuracy scores of all tests performed, including subject characteristics and/ or protein data, showed no evidence that a classifier based on a panel of all proteins and/or all characteristics could correctly classify the samples. The specificity and sensitivity of all characteristics was both 49%, of all proteins both 51%, and of a combination of all characteristics and proteins both 50%. We selected a panel containing characteristics and proteins with the best individual statistics and best RF importance factor score, consisting of body mass index (BMI), number of children, PRL and OPN. This panel resulted in improved average classification accuracy with sensitivity and specificity of both 59%.

Twenty-four women had been diagnosed with breast cancer as a result of a positive screening mammogram at the time of enrollment in the cohort. See Table S1 and S2 for the characteristics of these subjects and their samples. We hypothesized that in this group of cases the set of biomarkers tested could well be discriminative, but that their discriminative power might be masked when all samples are taken together in the classification analyses. Restriction of the analyses to the women in this subset did not change the results, however; again, cases could not be discriminated from matched controls (Figure 1B and Table S3).

### 2.3. Discussion

Using a serum panel of potential breast cancer markers consisting of OPN, haptoglobin, CA15-3, CEA, CA-125, prolactin, CA19-9, AFP, leptin and MIF, we were unable to predict the presence of early stage breast cancer. These proteins could not discriminate between the pre-diagnostic breast cancer samples and the control samples on their own, nor in combination with the other proteins, at least not in the samples and experimental set-up used by us.

This is the first study that investigated the predictive value of this combination of proteins that had individually been shown to be increased in blood samples from women diagnosed with breast cancer. A previous study by Kim *et al.* has shown that breast cancer markers with low sensitivity on their own, show increased sensitivity for breast cancer diagnosis when used in combination [19]. Four of the proteins included in our panel, CA15-3, CEA, CA-125 and prolactin, have earlier been investigated as tumor markers for breast cancer. In these case-control studies, they were found in slightly higher concentrations in women with symptomatic breast cancer [2,3,5–12,28]. Two other proteins in our panel, OPN and haptoglobin, were identified as very promising breast cancer biomarkers in mouse models for breast cancer, and were found to be higher in blood of women with breast cancer than in in that of controls [35,36,40]. MIF and leptin were previously found to be higher in breast cancer tissue than normal mammary/breast tissue [27,49], and CA19-9 and AFP were found to be related to other cancers before [2,23].

An important difference between these previous studies and ours is that we specifically designed our study for the detection of breast cancer in very early stages, by studying phase III serum samples that had been collected up to three years prior to the diagnosis of breast cancer. This “PRoBE (prospective-specimen collection, retrospective-blinded-evaluation) design” was first described by Pepe *et al*. [50]. The advantage is that any differences detected in biomarker levels are truly clinically relevant, because they can predict the presence of tumors in stages that are undetectable with detection techniques currently used [51]. However, the duration of the pre-diagnostic period of the samples collected in the current study may have been too long to detect differences for these markers. It is still possible that the proteins we selected to study do have predictive value for the presence of more advanced breast cancer, but apparently not up to three years prior to the detection on mammography or the occurrence of symptoms. One could question whether it is at all possible to detect increased marker levels in the blood at such early stages. In a previous study, however, using surface enhanced laser desorption/ionization time-of-flight mass spectrometry (SELDI-TOF MS) and two-dimensional nano-liquid chromatography coupled with tandem mass spectrometry (2D-nanoLC-MS/MS) for serum protein profiling, we were able to detect differences in the same pre-diagnostic sera of the same cases and controls as studied here [52], but these might be proteins involved in systemic responses rather than tumor derived markers. A recent study by Pitteri *et al.* also investigated pre-diagnostic breast cancer samples (plasma), using a quantitative analysis of acrylamide labeled proteins by LC-MS/MS. They detected elevated plasma levels of epidermal growth factor receptor up to 17 months before diagnosis [4].

We also focused specifically on a sub-group of cases with breast cancer detected following a positive screening mammogram at the time of enrollment in the cohort. In line with ovarian cancer, where based on increased CA-125 levels 83% of advanced stage ovarian cancers can be detected, but also 50% of patients with stage I disease [53], our set of ten markers might well have been discriminative in this specific subset of early breast cancer cases, but it was not.

A major advantage of the use of phase III serum samples is the elimination of collection bias. In studies investigating phase II specimens, major bias can be introduced due to systematic differences in the collection and handling of samples of cases and controls [15]. In the current nested case-control study, blood samples of all participants were collected, processed and stored in the exact same way, under strictly defined conditions, at a time when none of the participants had been diagnosed with breast cancer yet. This ensures that our results are not biased by differences in sampling handling between cases and controls [54]. In previous studies, sample handling has been shown to have a major impact on protein concentrations [55–61]. As suggested by Zhu *et al*., discovery studies should probably best be performed in pre-diagnostic samples, and subsequent validation of the detected potential markers also in pre-diagnostic samples [15].

Limitations of our study are its limited sample size and the fact that we do not have information on the estrogen receptor, progesterone receptor or HER2/neu status of the breast tumors. Because of lack of this information we are not able to separate the breast tumors by molecular subtype. It cannot be excluded that the heterogeneity among the breast cancers in our study is too large to identify a general predictive (combination) of markers. This might be especially a problem when the focus is on tumor-derived markers, such as CA-125, CA15-3, CA19-9, CEA, AFP OPN and haptoglobin. Markers may also perform better when longitudinal levels, obtained from serial samples, are evaluated [51,62].

## 3. Experimental Section

### 3.1. Study Population

We performed a case-control study nested within the Prospect-EPIC cohort. Prospect-EPIC is one of the two Dutch cohorts participating in the European Prospective Investigation into Cancer and nutrition, a large multicenter cohort study, including participants from ten European countries. From 1993 to 1997, 17,357 women from Utrecht and vicinity, then aged between 50 and 69 years, enrolled in the Prospect-EPIC cohort through the breast cancer screening program. Together with their invitation for the breast cancer screening, they received their invitation to participate in this cohort [48]. Participants filled out an extensive food frequency questionnaire and a general questionnaire. The latter contained questions on demographic characteristics, medical history, lifestyle characteristics and risk factors for cancer and other chronic diseases [48,63].

At enrollment, participants also donated a blood sample, which was routinely and identically processed and stored for all participants. Blood samples were stored in a climate-controlled refrigerator at 5 °C overnight. The next day, blood samples were centrifuged at 1500 g for 20 min and the serum was put in 0.5 mL straws. These straws were stored in a −86 °C freezer for about one week, until they were transported to liquid nitrogen tanks (−196 °C), where they have been stored ever since.

Participants were followed for vital and health status. Through the municipal registries, information on death and migration was obtained. Causes of death were obtained from the Central Bureau for Statistics (CBS). Through yearly linkage with the regional and national cancer registries, information about cancer incidence and stage of disease at diagnosis (tumor behavior, tumor size, lymph node involvement and metastasis) was obtained [48]. Until December 31st 2006, 687 women in the Prospect-EPIC cohort had been diagnosed with breast cancer. All participants signed an informed consent and the study was approved by the Institutional Review Board of the University Medical Center Utrecht.

For the current study, we selected women who were diagnosed with breast cancer within three years after enrollment in the cohort and who were postmenopausal then (no menstrual periods in the previous 12 months). Women were excluded if they had had cancer before, were suffering from diabetes, were current smokers or were currently using oral contraceptives or menopausal hormone therapy (HT). This was done to obtain a homogeneous group with respect to hormone levels, smoking status and metabolic status, because these factors may influence serum protein concentrations. Sixty-eight women were eventually included as a case. Controls were participants of the same cohort. We matched each case with one postmenopausal control that remained free of breast cancer, at least up to the time the case was diagnosed. Additional matching factors were age at enrollment (±1 year) and date of enrollment (±½ year). For controls the same exclusion criteria were applied as for cases.

### 3.2. Multiplex Analysis

The candidate breast cancer markers were analyzed with the use of three different kits. For OPN, leptin and MIF we used the Beadlyte^®^ Cancer Biomarker Panel kit (Millipore, St. Charles, MO, USA) and for CA15-3, CEA, CA19-9 and AFP the WideScreen^TM^ kit for Human Cancer Panel 1 (Novagen, Darmstadt, Germany) was used. CA-125 and prolactin were included in both kits. For haptoglobin we used the WideScreen™ kit for Human CVD Panel 5 (Novagen, Darmstadt, Germany). After retrieval of the serum samples from the liquid nitrogen, all samples had been thawed and aliquoted, and aliquots had been refrozen at −80 °C. These aliquots were later used for this analysis, meaning that all samples have been thawed twice.

The sample analysis was performed according to manufacturers’ instructions. In short, assays were carried out in 96-wells filter plates. Serum samples were diluted 1:6 for the Beadlyte^®^ kit, 1:5 for Widescreen™ panel 1 and 1: 5000 for Widescreen™ panel 5. Standard curve samples were prepared using serial dilution steps in a standard diluent. Standard curve samples provided quality control samples at two levels in the dynamic range of the standard curve. Serum samples and blanks were added to the plates randomly and blinded as to the case-control status of the sample. Standard curve samples and two quality control samples were applied to each plate in duplicate. Measurements were performed in singular, because of the limited amount of sample available. Fluorescently labeled, antibody-conjugated beads were applied to each well and incubated in the dark overnight at 4 °C for the Beadlyte^®^ plate, and 1 hour at room temperature (RT) for the WideScreen™ plates. Next, biotin-conjugated detection antibodies were added to the wells and incubated at RT in the dark (1.5 h for Beadlyte^®^ and 1 h for WideScreen™). Subsequently, Streptavidin-Phycoerythrin was applied to each well and incubated for 30 min at RT in the dark. After each incubation step, the plates were vacuumed and washed twice. Sheath fluid was applied to the wells of the Beadlyte^®^ plate, and a supplied buffer to the wells of the WideScreen™ plates. Finally, plates were shaken for 1–5 min in the dark and fluorescence intensity of the beads was analyzed on the Bio-plex instrument.

The quality of the assay was evaluated by the measurements of the quality control samples, which were within the expected concentration range provided by de manufacturer. The reproducibility of the measurements within a plate and between plates, was expressed by the CV of the measurements. The reproducibility was considered good if the CVs were smaller than 15%.

Values of samples below the standard curve were replaced with the lowest limit of quantification for that protein. In nine subjects (four cases and five controls), the measurements of one or more proteins were missing due to air bubbles or leaking wells. Due to the limited amount of serum available, these samples were not re-measured. Since removal of the values would mean loss of all analytes of the nine samples in multivariate analysis, we replaced the missing concentrations by imputed values using the *k*-nearest neighbor (KNN) procedure [64,65]. In this procedure, the missing value is imputed using nearest neighbor averaging. For each missing value, a value was imputed by averaging data from three subjects that were most similar with respect to the concentration of the other proteins, and for which that protein was not missing [64]

CA-125 and prolactin were measured both in the Beadlyte^®^ and the Widescreen™ kit. For CA-125 we used the Widescreen™ kit data, because with the Beadlyte^®^ data, in more than 50% of the samples the CA-125 concentration was below the detection range, possibly due to the higher dilution factor recommended for this assay. For prolactin we used the Beadlyte^®^ data for further analysis, because the Widescreen™ kit data contained four missing values due to technical issues. Since both assays showed high correlation (*r* = 0.88), this did not affect the overall analysis.

### 3.3. Data Analysis

Since the protein concentrations were not normally distributed, we applied a log-transformation to their values. To determine whether the mean log-transformed concentration of any of the candidate breast tumor markers was significantly different between pre-diagnostic serum samples of breast cancer patients and serum samples of healthy controls, we performed a paired samples *T* test using SPSS 15.0 (SPSS Inc.: Chicago, IL, USA). *P*-values <0.05 were considered statistically significant. We calculated geometric mean concentration and 95%CI for each protein in cases and controls, by taking the antilog of the mean and confidence limits on the transformed scale. For analysis of combinations of proteins, PCA and RF were applied. PCA was performed to identify principal components, which are linear combinations of the original variables that contain the largest variation between all samples studied. PCA was used here to examine whether the concentration values of all ten proteins combined could separate pre-diagnostic breast cancer patients and healthy controls [66,67]. For PCA analysis log transformed values of all ten proteins were imported into GenemathXT version 2.12 (Applied Math, Sint-Martens-Latem, Belgium). With RF analysis using all components, we investigated whether any combination of the proteins could discriminate between the cases and controls [68]. RF is an algorithm for classification that uses an ensemble of classification trees [68,69]. It has recently been used in several classification studies based on proteomic or genomic data, and is capable of identifying a classifier consisting of a subset of protein markers yielding the best predictive performance, *i.e.*, highest importance factor [70,71]. For the number of variables randomly sampled as candidates at each split, referred to as Mtry, the default value was chosen since similar prediction errors were obtained for different Mtry values. RF provides an importance factor for each protein that allows relative ranking of all proteins. To perform the RF analyses we used the scaled mean decrease in classification accuracy. Proteins were ranked according to their importance factor. To obtain stable estimates of the importance factor, large numbers of classification trees are needed [69,72]. RF does not overfit; therefore we performed the analyses with a large number of classification trees (40,000). In addition to protein data, we also investigated whether subject characteristics, or a combination of both proteins and characteristics, had any diagnostic value. For this, we performed three tests. All ten proteins were included in the analysis, characteristics included were BMI, age at menarche, age at menopause, former use of oral contraceptives, former use of hormone therapy, number of children, smoking habits, alcohol consumption, and level of education. The classification accuracy was calculated once on the basis of all proteins and/or characteristics, and once on the subset with the two best individual statistics (*i.e.*, best RF importance factor score). For this, the dataset was randomly split in a training set and a test set, where each set consisted of half the cases with their matched controls, this process was repeated 1000 times and the average classification accuracy score was calculated. RF analysis was performed in R program packages Random Forests [65,69].

Furthermore, we investigated whether the relations between the protein levels and breast cancer were stronger in subjects who were closest to diagnosis at the time of blood collection. To this end, we selected those cases who were diagnosed with breast cancer as the result of a positive screening mammogram at the time of enrollment, and we compared their protein concentrations with those of the matched controls with a paired sample *T* test. With PCA we tested whether the cases and controls in this subset could be separated, based on the concentrations of all proteins. RF was performed as described above.

## 4. Conclusions

Our results suggest that the tumor markers selected in the current study, single or in a panel, do not predict the presence of a very early stage of breast cancer. It cannot be excluded that the proteins may predict better in combinations with other proteins not selected here. It could also be that they predict better for specific molecular subtypes of breast cancer. Future studies should therefore preferentially select a broader target set of potential biomarkers, which could be enabled by new technologies based on antibody arrays that can measure up to 100 proteins in small amounts of serum [73], and include sufficient numbers of the different molecular tumor subtypes so that a distinct predictive combination of biomarkers for each subtype could be identified.

## Supplementary Materials



## Figures and Tables

**Table 1:** List of biomarkers tested.

Biomarker	Rationale
CA15-3	Monitoring marker breast cancer a [2,12]
CEA	Monitoring marker breast cancer a [2,12]
CA-125	Monitoring marker breast and ovarian cancer a [11]
CA19-9	Monitoring marker pancreatic and gastrointestinal cancer a [2,23] Increased serum levels in human breast cancer cases b [19]
AFP	Staging marker testis, ovary and liver cancer a [2] Increased serum levels in human breast cancer cases b [22]
MIF	Higher expression in mammary tumors compared to normal tissue [27] Increased serum levels in human breast cancer cases b [20,21]
Prolactin	Risk marker for breast cancer a [28]
Leptin	Higher expression in mammary tumors compared to normal tissue [49] Risk marker for breast cancer [29] Differential plasma levels in human breast cancer cases a [30–32,40]
OPN	Higher in humanized Mouse models for breast cancer [35] Increased plasma [40] and serum [37] levels in human breast cancer cases b Increasing expression in mammary tumor tissue [41–43] and plasma [44,45] in progressing disease
Haptoglobin	Higher in humanized Mouse models for breast cancer [35] Increased serum levels in human breast cancer cases b [36]

OPN: osteopontin; CA15-3: cancer antigen 15-3; CEA: carcinoembryonic antigen; CA-125: cancer antigen 125; CA19-9: cancer antigen 19-9; AFP: α-fetoprotein; MIF: migration inhibitory factor.

a Higher in advanced disease;

b Compared to healthy controls.

**Table 2:** Study population characteristics.

	Cases (*n* = 68)	Controls (*n* = 68)
**Age at enrollment** (years)		
Mean ± SD	60.2 ± 5.6	60.3 ± 5.7
**Age at menarche** (years)		
Mean ± SD	13.4 ± 1.6	13.7 ± 1.9
Missing	1	2
**Age at menopause** (years)		
Mean ± SD	49.0 ± 5.6	49.0 ± 5.3
Missing	-	3
**BMI**		
Mean ± SD	26.6 ± 3.1	26.3 ± 3.6
Missing	1	-
**Use of oral contraceptives**, *n* (%)		
No, but used to in the past	36 (52.9)	40 (58.8)
No, never	32 (47.1)	28 (41.2)
**Duration of oral contraceptives use** a (years)		
Median (IQR)	10.0 (4.0–16.0)	4.5 (2.0–10.0)
**Use of HT**, *n* (%)		
No, but used to in the past	7 (10.3)	6 (8.8)
No, never	61 (89.7)	62 (91.2)
**Duration of HT use** a (years)		
Median (IQR)	1.0 (1.0–8.0)	2.0 (1.0–10.0)
**Ovariectomy**, *n* (%)		
Both ovaries removed	5 (7.4)	3 (4.4)
Missing	-	1
**Parity**, *n* (%)		
Nulliparous	10 (14.7)	5 (7.4)
**Number of children** b		
Median (IQR)	2 (2–3)	3 (2–3)
**Smoking**, *n* (%)		
No, but used to in the past	31 (45.6)	34 (50.0)
No, never	37 (54.4)	34 (50.0)
**Pack-years smoking until stop date** c		
Median (IQR)	7.9 (1.9–16.4)	4.1 (1.4–10.2)
Missing	1	3
**Alcohol intake** d (g/day)		
Median (IQR)	2.0 (0.2–7.2)	2.5 (0.2–8.4)
**Highest level of education** e, *n* (%)		
Level 1	35 (51.5)	37 (54.4)
Level 2	21 (30.9)	18 (26.5)
Level 3	12 (17.6)	13 (19.1)

SD: standard deviation; BMI: body mass index; IQR: inter-quartile range; HT: menopausal hormone therapy.

a Among former oral contraceptives/HT users;

b Among women with children;

c Among former smokers;

d Energy-adjusted alcohol intake at enrollment;

e Level 1 = primary education or lower vocational education, Level 2 = advanced elementary education or intermediate vocational education, Level 3 = higher general secondary education, higher vocational education or university.

**Table 3:** Characteristics of the serum samples.

	Cases (*n* = 68)	Controls (*n* = 68)
**Serum sample storage duration** a (years)		
Mean ± SD	13.6 ± 1.1	13.5 ± 1.1
**Hours in refrigerator** b		
Median (IQR)	22 (21–23)	22 (20–23)
**Days at −86 °C** c		
Median (IQR)	8 (6–11)	7 (5–11)
**Subjects use of medicines, minerals or vitamins last week** d, *n* (%)		
Yes	46 (67.6)	44 (64.7)
No	22 (32.4)	24 (35.3)
**Time since last meal and/or drink of subject** d (minutes)		
Median (IQR)	108 (87–137)	116 (88–137)

SD: standard deviation; IQR: inter-quartile range.

a Until experiment;

b Between collection and centrifugation;

c Between centrifugation and storage at liquid nitrogen;

d At moment of blood collection.

**Table 4:** Serum concentrations of all measured proteins in the pre-diagnostic breast cancer- and healthy control samples.

	Cases (*n* = 68)		Controls (*n* = 68)		Paired *T* test

Biomarker	Geometric mean concentration	95%CI	Geometric mean concentration	95%CI	*P*-value
OPN (ng/mL)	1.11	0.86–1.44	1.17	0.83–1.66	0.82
Haptoglobin (mg/mL)	1.10	0.86–1.39	1.08	0.87–1.33	0.89
CA15-3 (U/mL)	8.48	7.08–10.15	10.34	8.72–12.26	0.11
CEA (ng/mL)	0.56	0.48–0.66	0.49	0.40–0.60	0.29
CA-125 (U/mL)	2.19	1.64–2.92	1.95	1.45–2.63	0.56
Prolactin (ng/mL)	4.79	4.09–5.60	4.03	3.48–4.66	0.10
CA19-9 (U/mL)	2.30	1.74–3.02	2.10	1.57–2.79	0.66
AFP (ng/mL)	0.50	0.40–0.64	0.46	0.36–0.59	0.62
Leptin (ng/mL)	17.34	14.38–20.92	15.67	12.91–19.02	0.43
MIF (pg/mL)	166.5	141.9–195.4	199.7	165.9–240.4	0.13

95%CI: 95% confidence interval; OPN: osteopontin; CA15-3: cancer antigen 15-3; CEA: carcinoembryonic antigen; CA-125: cancer antigen 125; CA19-9: cancer antigen 19-9; AFP: α-fetoprotein; MIF: migration inhibitory factor.
