# Supplementary material for: A Bead-Based Multiplexed Immunoassay to Evaluate Breast Cancer Biomarkers for Early Detection in Pre-Diagnostic Serum
Source: Int J Mol Sci. 2012 Oct 22;13(10):13587–604. doi: 10.3390/ijms131013587 (PMC3497343; doi:10.3390/ijms131013587)
Supplement: Supplementary file 1 [file ijms-13-13587-s001.pdf]

Supplementary Information

Table S1. Characteristics of the subset.

	**Cases** (*n* = 24)	**Controls** (*n* = 24)
**Age at enrollment** (years)		
Mean ±SD	60.9 ± 5.2	60.9 ± 5.3
**Age at menarche** (years)		
Mean ±SD	13.7 ± 1.5	13.4 ± 1.7
Missing	1	2
**Age at menopause** (years)		
Mean ±SD	49.4 ± 6.9	50.0 ± 4.6
Missing	-	1
**BMI**		
Mean ±SD	27.7 ± 3.4	26.5 ± 4.2
Missing	1	-
**Use of oral contraceptives**, *n* (%)		
No, but used to in the past	12 (50.0)	11 (45.8)
No, never	12 (50.0)	13 (54.2)
**Duration of oral contraceptives use** [a] (years)		
Median (IQR)	10 (4–15)	6 (2–10)
**Use of HT,** *n* (%)		
No, but used to in the past	2 (8.3)	4 (16.7)
No, never	22 (91.7)	20 (83.3)
**Duration of HT use** [a] (years)		
Median (IQR)	1.0 (4.5–8.0)	1.5 (1.0–8.0)
**Ovariectomy**, *n (%)*		
Both ovaries removed	1 (4.2)	-
**Parity**, *n* (%)		
Nulliparous	1 (4.2)	-
**Number of children** [b]		
Median (IQR)	2 (2–3)	3 (3–4)
**Smoking**, *n* (%)		
No, but used to in the past	12 (50.0)	10 (41.7)
No, never	12 (50.0)	14 (58.3)
**Pack-years smoking until stop date** [c]		
Median (IQR)	3.8 (1.7–9.6)	3.4 (0.9–12.8)
Missing	1	1
**Alcohol intake** [d] (g/day)		
Median (IQR)	3.0 (0.4–9.0)	0.5 (0.0–4.6)
**Highest level of education** [e], *n* (%)		
Level 1	11 (45.8)	15 (62.5)
Level 2	13 (54.2)	6 (25.0)
Level 3	0 (0)	3 (12.5)

SD: standard deviation; BMI: body mass index; IQR: inter-quartile range; HT: menopausal hormone therapy. [a] Among former oral contraceptives/HT users. [b] Among women with children. [c] Among former smokers. [d] Energy-adjusted alcohol intake at enrollment. [e] Level 1 = primary education or lower vocational education, Level 2 = advanced elementary education or intermediate vocational education, Level 3 = higher general secondary education, higher vocational education or university.

Table S2. Characteristics of the serum samples in the subset.

	**Cases (n = 24)**	**Controls (n = 24)**
**Serum sample storage duration** [a] (years)		
Mean ± SD	11.4 ± 1.3	11.4 ± 1.3
**Hours in refrigerator** [b]		
Median (IQR)	22 (20–23)	22 (19–23)
**Days at −86 °C** [c]		
Median (IQR)	8 (5–21)	7 (5–21)
**Subjects use of medicines, minerals or vitamins last week** [d], n (%)		
Yes	17 (70.8)	14 (58.3)
No	7 (29.2)	10 (41.7)
**Time since last meal and/or drink of subject** [d] (minutes)		
Median (IQR)	122 (95–160)	131 (90–140)

SD: standard deviation; IQR: inter-quartile range. [a] Until experiment. [b] Between collection and centrifugation. [c] Between centrifugation and storage at liquid nitrogen. [d] At moment of blood collection

Table S3. Serum concentrations of all measured proteins in subset of cases diagnosed at 1st screening after inclusion and matched healthy controls.

	**Cases (n = 24)**		**Controls (n = 24)**		**Paired T test**
**Biomarker**	**Geometric mean concentration**	**95%CI**	**Geometric mean concentration**	**95%CI**	**p-value**
OPN (ng/mL)	1.21	0.80–1.82	1.18	0.65–2.15	0.96
Haptoglobin (mg/mL)	1.34	0.91–1.96	1.10	0.75–1.62	0.41
CA15-3 (U/mL)	7.82	5.83–10.49	9.02	6.72–12.11	0.45
CEA (ng/mL)	0.57	0.43–0.75	0.44	0.32–0.60	0.27
CA-125 (U/mL)	1.99	1.17–3.39	1.33	0.77–2.30	0.28
Prolactin (ng/mL)	3.66	3.05–4.38	3.57	2.57–4.97	0.90
CA19-9 (U/mL)	1.83	1.16–2.89	2.19	1.50–3.21	0.53
AFP (ng/mL)	0.37	0.24–0.57	0.41	0.27–0.63	0.69
Leptin (ng/mL)	21.89	17.02–28.15	18.45	12.91–26.37	0.39
MIF (pg/mL)	145.3	101.8–207.5	204.1	146.2–284.9	0.15

95%CI: 95% confidence interval; OPN: osteopontin; CA15-3: cancer antigen 15-3; CEA: carcinoembryonic antigen; CA-125: cancer antigen 125; CA19-9: cancer antigen 19-9; AFP: α-fetoprotein; MIF: migration inhibitory factor.

© 2012 by the authors; licensee MDPI, Basel, Switzerland. This article is an open access article distributed under the terms and conditions of the Creative Commons Attribution license (http://creativecommons.org/licenses/by/3.0/).
